# New Evidence of Renal and Cardiovascular Alterations Promoted by Bisphenol A

**DOI:** 10.3390/biom11111649

**Published:** 2021-11-08

**Authors:** Rafael Moreno-Gómez-Toledano, María I. Arenas, Esperanza Vélez-Vélez, Marta Saura, Ricardo J. Bosch

**Affiliations:** 1 Universidad de Alcalá, Department of Biological Systems/Physiology, IRYCIS, 28871 Alcalá de Henares, Spain; marta.saura@uah.es (M.S.); ricardoj.bosch@gmail.com (R.J.B.); 2 Universidad de Alcalá, Department of Biomedicine and Biotechnology, 28871 Alcalá de Henares, Spain; misabel.arenas@uah.es; 3 Fundación Jiménez Díaz School of Nursing, Jiménez Díaz Foundation, Autonomous University of Madrid, 28040 Madrid, Spain; evelez@fjd.es

Bisphenol A (BPA) is a phenolic compound that is widely used to synthesize plastics as a monomer or additive [1]. The unbeatable quality–price relationship has made plastics the central axis of contemporary life, which is why BPA can be found in many articles for food, cosmetic, or even textile use [2,3,4,5]. BPA is a well-known molecule that has been widely investigated for its properties as an endocrine disruptor, capable of exerting pro-estrogenic and anti-androgenic effects [6,7]. Numerous contributions have emerged in the last two decades linking BPA to pathologies such as diabetes, hypertension, obesity, kidney disease, cancer, and even behavioral disorders [8,9,10,11,12,13,14].

Previous work by our team has observed that BPA could induce apoptosis and dysregulation of structural proteins in cultured mouse podocytes (a highly specialized cell type that forms part of the glomerular filtration barrier, responsible for correct filtering of blood in the renal corpuscle). Furthermore, its intraperitoneal administration in mice induced renal and structural function changes similar to those observed in the early stages of diabetic nephropathy (DN), such as albuminuria, glomerular hyperfiltration, mesangial expansion, and podocytopenia [15]. New evidence suggests that patients with kidney disease release podocytes in their urine, and on numerous occasions, these are viable [16,17]. For this reason, the adhesion mechanisms of human podocytes in culture treated with low doses of BPA were studied, observing a critical detachment and downregulation of numerous structural and adhesion proteins [18]. In parallel, other previous works by our team also demonstrated that BPA administration in drinking water could induce hypertension in a dose-dependent manner, a process related to the uncoupling of the enzyme endothelial nitric oxide synthase [19]. The mechanism of BPA-induced hypertension, regulated by the enzyme calcium-calmodulin dependent protein kinase II, was closely related to necroptosis, a novel mechanism of cell death [20].

Recently, thanks to the Special Issue “Bisphenol A: An Environmental Factor with an Emerging Role in the Pathophysiology of Renal and Cardiovascular Diseases” published in *Biomolecules*, new contributions have been compiled that reinforce the position of BPA as an environmental factor involved in the development or progression of renal and cardiovascular diseases.

Firstly, in Kobroob et al. [21], the authors verified that the kidney damage induced by BPA may be related to oxidative stress, and the administration of an antioxidant such as N-acetyl cysteine (NAC) is capable of restoring mitochondrial integrity and oxidative balance. Interestingly, the authors used a relatively low dose, since the 50 mg/kg dose has been considered no observable effect level (NOEL) in the renal system [22,23,24]. Despite this, treatment at 16 weeks with BPA was able to induce important changes in kidney function. Body weight and food intake were lower with BPA and NAC + BPA, despite BPA being considered an obesity-related molecule [25,26]. A smaller kidney size was also observed, although no significant changes were observed in the renal hypertrophy index when adjusting for the animal’s weight. In the functional aspects of the kidney, treatment with BPA induced a notable and significant increase in urea nitrogen, serum creatinine, 24-hour proteinuria, and the urine protein-to-creatinine ratio, as well as a significant reduction in the clearance of creatinine. Curiously, cotreatment with NAC ameliorated the pathological changes induced by BPA. Similarly, histology showed significant atrophy in the renal corpuscle and many apoptotic cells distributed throughout the renal tubules. Again, cotreatment with NAC significantly alleviated BPA-induced damage. In addition, significant increases in molecules related to oxidative stress were also observed, and considerable reductions in the expression of antioxidant enzymes could be reversed with NAC. Finally, the authors observed that the kidney damage induced by BPA and alleviated by NAC is related to the AMPK-PGC-1α-SIRT3-SOD2 signaling pathway, which is involved in mitochondrial homeostasis.

Interestingly, the oxidative stress induced by low doses of BPA was also studied by Moreno-Gómez-Toledano et al. [27]. The authors used doses of BPA below the cytotoxic threshold and found that BPA was able to promote cell senescence in a primary mouse vascular endothelial cell line. After identifying senescence-inducing concentrations by the β-gal assay, they confirmed that the expression of the senescence markers p16 and p21 only increased to 5 µM. Since oxidative stress is one of the elements responsible for cellular senescence [28], carbamylated proteins and superoxide anion production were analyzed, confirming significant increases at the 5 µM concentration. Next, the relative expression levels of mRNAs related to the unfolding protein response (UPR) system were analyzed using qPCR, since this is related to cell senescence [29]. The results showed that BPA could induce a significant increase in the PERK-ATF4-CHOP pathway, the only one of the three signaling pathways of the UPR system related to pathological processes. In line with the cellular studies, an animal model was carried out with half the NOEL concentration (25 mg/kg) in drinking water. Administration of the compound via this route maintains a relatively constant and natural entry of BPA into the body, which ensures chronic exposure. Analogous to the results observed in endothelial cells in culture, the vascular endothelium of the animals showed a significant increase in the senescence proteins p16 and p21 and the CHOP protein.

Finally, to verify that the acceleration of cellular aging induced by BPA is related to oxidative stress, a new cellular study was carried out, in which NAC was used. In the β-gal assay, it was shown that cotreatment with NAC not only prevented BPA-induced senescence but even reduced it below the level of the control group. Western blot analysis confirmed that the relative expression levels of the p16 and p21 proteins were similar to those of the control group. Finally, the proteins of the UPR system were also expressed similarly to the control group after cotreatment with NAC.

Another work highlighted in this Special Issue is Moreno-Gómez-Toledano et al. [30]. In this case, by conducting complete statistical work, they found new evidence that correlates BPA with human kidney disease. The first part of the work focused on unifying all the studies within the BPA–kidney disease paradigm to perform a combined meta-analysis. The different characteristics of the studies identified by systematic review made it impossible to develop a single meta-analysis. Therefore, several studies were carried out in parallel, unifying studies with similar characteristics. First, the combined analysis of BPA in the blood and risk of chronic kidney disease (CKD) showed a robust and positive result. In the urinary BPA study, the analyses related to albuminuria and low-grade albuminuria did not show conclusive results, although positive trends were shown. However, the combined analysis of the studies related to urinary BPA and renal function (estimated glomerular filtration rate) showed that increases in urinary BPA were related to a decrease in renal function (higher CKD risk).

The second part of the work analyzed the available data from the American National Health and Nutrition Examination Survey (NHANES) cohort, comparing urinary BPA values with renal function. Thus, it was observed that individuals with a higher concentration of urinary BPA had higher concentrations of albumin in their urine, although the expected relationship with the estimated renal function was not found. Similarly, when analyzing individuals with low-grade albuminuria, a significant increase in urinary BPA was shown. Finally, when studying the urinary BPA value of individuals diagnosed with kidney disease, a significant increase was determined, which was even higher in those undergoing dialysis treatment.

In conclusion, the new evidence supports and reinforces the hypothesis that BPA is an environmental factor related to renal and cardiovascular diseases.
